# Nasopharyngeal and Peripheral Blood Type II Interferon Signature Evaluation in Infants during Respiratory Syncytial Virus Infection

**DOI:** 10.3390/medicina60020259

**Published:** 2024-02-02

**Authors:** Francesco Savino, Maddalena Dini, Anna Clemente, Cristina Calvi, Anna Pau, Ilaria Galliano, Stefano Gambarino, Massimiliano Bergallo

**Affiliations:** 1 Early Infancy Special Care Unit, Regina Margherita Children Hospital, A.O.U. Città della Salute e della Scienza di Torino, 10126 Torino, Italy; francesco.savino@unito.it; 2 Paediatric Laboratory, Department of Public Health and Pediatric Sciences, Medical School, University of Turin, 10136 Turin, Italy; maddalena.dini@unito.it (M.D.); anna.clemente424@edu.unito.it (A.C.); cristina.calvi@unito.it (C.C.); anna.pau@edu.unito.it (A.P.); ilaria.galliano@unito.it (I.G.);; 3 BioMole srl, Via Quarello 15/A, 10135 Turin, Italy; 4 Department of Pediatrics, Infectious Diseases Unit, Regina Margherita Children’s Hospital, University of Turin, Piazza Polonia 94, 10126 Turin, Italy

**Keywords:** type II IFN signature, IFNγ, RSV, infants, PCR real time

## Abstract

*Background and Objectives:* In this study, we applied one-step real time rt-PCR technology type II INF signature to blood and nasopharyngeal (NPS) swabs of acute early recovery children < 1 years hospitalized for bronchiolitis with laboratory-confirmed RSV infection. *Materials and Methods:* A prospective observational case–control study was conducted in 2021–2022. The study took place in Children Hospital “Regina Margherita”, Torino Italy. The study included 66 infants, of which 30 patients were hospitalized for bronchiolitis due to RSV infection and 36 age-matched controls. Inclusion criteria included a positive RSV test for infants with bronchiolitis. We collected peripheral blood and nasopharyngeal swabs for relative quantification of type II Interferon signature by One-Step Multiplex PCR real time. *Results:* IFN levels were downregulated in the peripheral blood of bronchiolitis patients; these data were not confirmed in the nasopharyngeal swab. There was no correlation between NPS and the type II IFN score in peripheral blood. *Conclusions:* our study shows for the first time that type II IFN score was significant reduced in peripheral blood of infants with bronchiolitis by RSV compared to age-matched healthy controls; in the NPS swab this resulted downregulation was not statistically significant and the type II IFN score in the NPS swab can be used as marker of resolution of infection or improvement of clinical conditions.

## 1. Introduction

Respiratory syncytial virus (RSV) is the main cause of hospitalization for bronchiolitis among infants younger than 12 months [1]. Worldwide, RSV causes almost 34 million lower respiratory tract infections (LRTI) with an estimated annual increase of 10% and 3.4 million hospitalizations per year in infants and children less than 5 years of age [2,3]. In numerous nations, including Italy, RSV currently represents a public health issue [4]. RSV belongs to the Pneumoviridae family [5] and is characterized by a large envelope and a negative-sense RNA (approximately 15–16 kb) encoding for 11 proteins, which include both non-structural and structural proteins. It owes its name to its ability to form syncytia from neighboring cells of the host after infection with the virus. It is an RNA virus with a linear, single-stranded genome surrounded by a helical nucleocapsid, which in turn is surrounded by a lipoprotein envelope that gives it a spherical or filamentous appearance. Among the latter, the membrane glycoproteins G and F should be emphasized, which mediate adhesion and fusion with the surface of the epithelial cells of the respiratory tract. Glycoprotein F is also involved in the formation of the characteristic syncytia. The structural proteins also include the matrix protein (M), which is involved in the assembly of the virus, two nucleocapsid proteins (N and P) and the proteins M2-1 and M2-2, which are responsible for transcriptional activity and regulation. RSV also has an RNA-dependent RNA polymerase (L) that controls transcription and replication of the virus in the host cell cytoplasm once the virus has entered. The non-structural proteins NS1 and NS2 are the first to be transcribed during infection and interfere with the interferon (IFN) response and other elements of the immune system. RSV infections show seasonality, with peaks through the winter months in temperate regions [6,7]. The elderly, young children, and those with chronic medical conditions are at the highest risk for severe RSV infections [8,9]. The initial RSV infection progresses to affect the lower respiratory tract and within 2–3 days 25–30% of children develop acute bronchiolitis. The initial picture of a runny nose and cough develops into persistent cough, progressive increase in work of breathing, severe deterioration and refusal to feed. Clinical signs (tachypnoea, dragging, nosebleeds, widespread wheezing, thoracic hyperinflation, generalized hypoventilation, hypoxaemia and cyanosis) and radiological signs (air trapping, areas of consolidation or serious complications such as pneumonia and atelectasis) characteristic of severe bronchopulmonary involvement become evident on examination. The development of acute bronchiolitis is unpredictable at the onset of the disease: most infants with acute RSV bronchiolitis who were previously healthy and had no pulmonary complications improve within 3–4 days without the need for hospitalization; of those who are hospitalized, many improve with symptomatic treatment and oxygen therapy and can be discharged after 2–3 days. On the other hand, 1–3% of the youngest infants (under 6 months, especially under 2 months) and children with underlying conditions usually develop pulmonary complications. They require a longer hospital stay and are often admitted to the pediatric or neonatal intensive care unit to support breathing and treat the developing respiratory complications (pneumonia, pneumothorax, atelectasis). However, there is some evidence that the ability to develop an adequate type I-like immune response during primary RSV infection is impaired in the development of severe lower respiratory tract disease [10].

Although IFN type II, known as IFN-γ, has a similar nomenclature to IFN type I, it is signaled via a different receptor, has effects that are independent of IFN type I and is mainly produced by natural killer cells during infection (2 IFN-γ was originally identified 30 years ago as an antiviral agent and has since been characterized as a homodimeric glycoprotein with pleiotropic immunological functions). IFN-γ is primarily secreted by activated T cells and natural killer (NK) cells and can promote macrophage activation, mediate antiviral and antibacterial immunity, enhance antigen presentation, regulate innate immune activation, coordinate lymphocyte–endothelial interaction, regulate Th1/Th2 balance, and control cell proliferation and apoptosis. Only 20 years after the identification of IFN-γ was its cell surface receptor discovered. The α-chain of IFN-γR, also known as IFN-γR1 or CD119, was the first component of the receptor to be identified and cloned. Although it binds IFN-γ with relatively high affinity, IFN-γR1 alone is unable to mediate the biological responses to this cytokine. Other studies led to the identification and cloning of IFN-γR2, as the protein required in addition to IFN-γR1 to endow a cell with the ability to respond to IFN-γ. Specific residues within the cytoplasmic domains of both the α and β chains of IFN-γR are critical for the transduction of IFN-γ signaling from the cell surface to the nucleus through the activation of intracellular signaling pathways [11,12,13]. The IFN-γ is the sole IFN released by NK cells and mostly by Th1 cells, which also elicits activation of thousands of genes [14]. CXCL9, CXCL10, and IDO1 are prevalently IFN-γ-stimulated genes (ISG). Their expressions correlate with the tissue infiltration of inflammatory cells, in particular of T cells [15]. Downregulation of IFN-γ were reported in children with Influenza Virus illness, SARS-CoV2 and RSV bronchiolitis [16,17,18]. IFN-γ has immunoregulatory functions that optimize the antiviral response and limit exaggerated responses that could lead to collateral damage. An optimal antiviral response involves both the activation of beneficial immune responses and the simultaneous inhibition of inappropriate and potentially harmful responses [19].

Several RSV transcriptome studies have been performed using in vitro models [20,21,22,23,24,25,26], animal models [27,28,29,30,31] and human subjects [32,33,34,35,36,37,38]. However, most in vivo studies only investigated systemic transcriptional profiles in blood [33,34,35,37,38]. Only one study investigated local respiratory expression profiles by analyzing nasopharyngeal swabs (NPS) from hospitalized children (*n* = 30) [39].

In this study, we applied one-step real time rt-PCR technology type II INF signature to blood and nasopharyngeal (NPS) swabs of acute early recovery children < 1 years hospitalized for bronchiolitis with laboratory-confirmed RSV infection.

## 2. Materials and Methods

### 2.1. Patients

In this study we enrolled full-term infants who were hospitalized in the Early Infancy Special Care Unit of the Regina Margherita Children Hospital, Turin, Italy, for their first episode of bronchiolitis between October 2022 and February 2023. The controls were healthy full-term infants below 12 months of age who attended an outpatient clinic at the Department of Pediatrics for routine postnatal checks.

Bronchiolitis was diagnosed by using clinical signs that included rales, wheezing with or without a cough, dyspnea and retractions of the respiratory muscles and increased respiratory rate. The hospitalized infants with bronchiolitis underwent routine blood and swab tests at their recovery in Hospital.

The parents of the enrolled infants (subject and control) were informed about the purpose and benefits of the study, and written, informed consent was obtained. The protocol was approved by the Ethics Committee of the Azienda Ospedaliera Città della Salute e della Scienza di Turin, Italy date: 24 November 2014 prot. 116918.

The mean age of the 30 bronchiolitis patients (46% male and 54% female) was 86 days (10–351) when they were admitted to hospital. Their mean gestational age at birth was 38 weeks and their mean birthweight was 3150 g (2690–3910). The 36 infants in the control group (51.6% boys) were seen at a mean age of 92 days (22–333) and 43.2% were still being exclusively or predominantly breastfed. They had not been hospitalized for bronchiolitis or any other infections. In the 30 bronchiolitis subjects, the mean gestational age at birth was 38 weeks and their mean birthweight was 3020 g (2500–3880). White blood cells, neutrophils and eosinophils and RSV positivity were recovered from the medical records. All the samples were screened for other respiratory viruses with Allplex Respiratory Panel Assays (Seegene Inc. Taewon Building, Seoul, Republic of Korea) and resulted negative.

In brief, at admission (acute phase) blood was collected in ethylenediaminetetraacetic acid (EDTA), and RNApro (BioMole) and Nasopharyngeal swabs (NPS) (Copan Diagnostics Inc., Murrieta, CA, USA) were collected in RNApro (BioMole). NPS and blood were processed for host gene expression profile analyses. NPS recovered from healthy subjects were 20 out of 30.

The exclusion criteria for all samples included premature births under 37 weeks’ gestation, known or suspected impairment of immune function and congenital malformations. A pediatric investigator recorded the personal data provided by the parents or guardians and the clinical data collected during the physical examinations.

### 2.2. mRNA Isolation and Real-Time PCR

For each nasopharyngeal swab and peripheral blood, RNA was extracted using simply RNA Blood Kit protocol in Maxwell16 system (Promega, Madison, WI, USA), according to the manufacturer’s instructions. Prior to extraction, swabs and peripheral blood were maintained in RNApro (BioMole), a stabilizer that permits conservation of the samples in −80 °C until use without RNA degradation. RNA was eluted in a final volume of 50 μL. RNA purity and concentration were evaluated by spectrophotometry using Simplinano (Biochrom, Cambridge, UK). Absorbance ratios of 260/280 were used to assess the purity of nucleic acid extracted.

Relative quantification of the type II IFN signature was achieved by IFNsig. Type II One-Step Multiplex PCR real time kit was used. BM-024 (BioMole, Turin, Italy). Amplifications were run in CFX96 Real-Time System (Bio-Rad Laboratories, Segrate Milan, Italy) using Maestro software ver. 1.0. The IFNsig.Type II Multiplex One Step kit is a real-time PCR assay able to reverse-transcribe and amplify RNA in a single step. Thanks to the multiplex version, the RNA is tested with two different mixes that will return the data of the genes stimulated by interferon-γ and the housekeeping gene.

10 ng of RNA was amplified in a 20 μL total volume reaction. The amplifications were performed in a 96-well plate at 50 °C for 10 min, 95 °C for 10 min, followed by 40 cycles of 95 °C for 10 s and 60 °C for 30 s for a total time of 80 min.

### 2.3. IFN Signature Analysis

The expressions of four IFN-stimulated genes were assessed by qPCR using CFX96 Real Time PCR System (BioRad, Hercules, CA, USA) and IFNsig. Type II One-Step Multiplex PCR real time kit (BioMole) for IFN-γ, CXCL9, CXCL10 and IDO1 was used [15]. Each target quantity was normalized with the expression level of GAPDH, and the relative quantification (RQ) was conducted relating to 30 healthy controls using the 2^−ΔΔCt^ method [40]. The median fold change of the four genes was used to calculate the IFN score for each subject. The kit also contained a positive control (Quantirel) that permits monitoring of all the procedure steps.

### 2.4. Statistical Analysis

Statistical analyses were performed using GraphPAD Prism5 (GraphPad Software, La Jolla, CA, USA). We used the non-parametrical Mann–Whitney U-Test to compare the IFN-score in the analyzed patient groups and controls. We used the Spearman correlation test comparing IFN scores between NPS swab and blood. We considered a difference to be significant if the *p* value was <0.05.

## 3. Results

### 3.1. Study Populations

At enrollment, all the patients were screened for the RSV infection using rapid Antigen Xpert Xpress FLU/RSV (Cepheid, Sunnyvale, CA, USA): 30 infants suffering from bronchiolitis were positive for RSV infections and 36 healthy controls were negative. No coinfection was detected.

White blood cells count (WCC) were higher in the RSV group, but not significantly. However, lymphocytes and monocytes were significantly higher in the RSV group than the healthy controls (Table 1).

### 3.2. IFN-Stimulated Genes Expression Evaluated by qPCR

**Figure 1:**
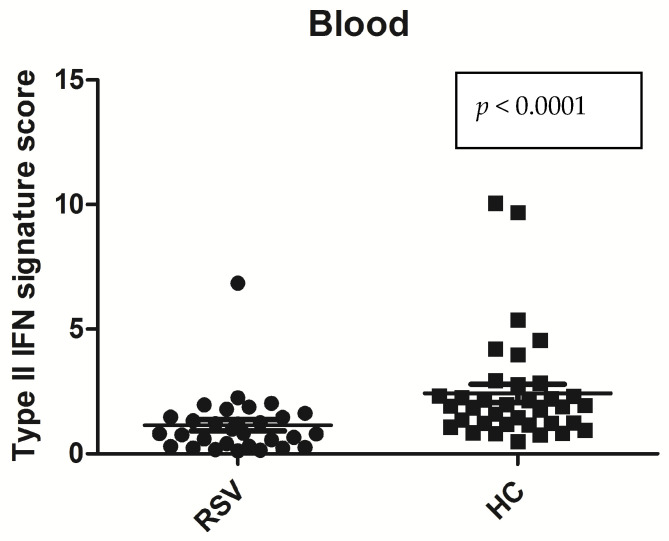
Statistical analysis: Mann–Whitney test was used to compare IFN score in the peripheral blood of bronchiolitis RSV patients vs. healthy control. Circles and squares show IFN score of bronchiolitis and healthy controls, horizontal lines show the median values.

As a first attempt, we investigated type II IFN signature in blood samples. We calculated the IFN score for each sample as shown in Figure 1 and Table 2 and Table 3. The IFN scores were significantly higher in the HC than in the bronchiolitis subject. Mean value: IFN score RSV positive bronchiolitis samples: 1.14 ± 1.25 vs. mean value: IFN score healthy controls: 2.41 ± 2.14 (*p* < 0001).

**Figure 2:**
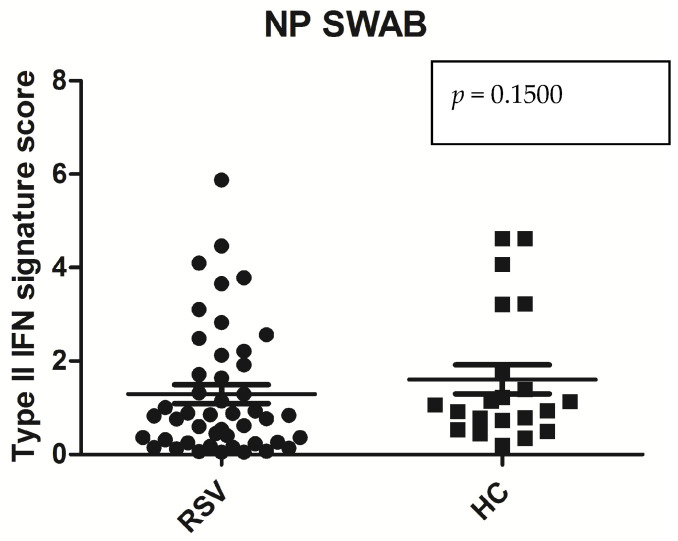
Statistical analysis: Mann–Whitney test was used to compare IFN score analyzed in NPS swab of bronchiolitis RSV patients vs. healthy control. Circles and squares show IFN score of bronchiolitis and healthy controls, horizontal lines show the median values.

As a second attempt, we investigated the type II IFN signature in the NPS swab samples. We calculated the IFN score for each sample as show in Figure 2. The IFN scores were higher but not statistically significant in the HC than in the bronchiolitis subject. Mean value: IFN score RSV positive bronchiolitis samples: 1.29 ± 1.37 vs. mean value: IFN score healthy controls: 1.55 ± 1.45 (*p* = 0.15).

**Figure 3:**
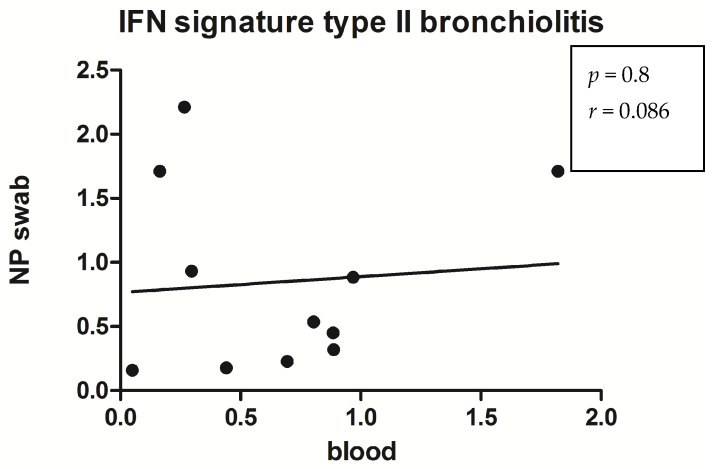
Spearman correlation test was used to compare IFN score analyzed in NPS swab and peripheral blood of bronchiolitis patients.

We calculated the IFN score correlation analysis between NPS and blood, not trying to find it to be statistically significant with *p* = 0.8 (Figure 3) and Table 4 and Table 5.

## 4. Discussion

RSV is, in infancy, the most important etiological agent of acute lower respiratory tract infections, and the leading cause of hospitalization in childhood, which results in a great problem for global health-care services as well as a significant pathogen for the elderly [41]. It is estimated that 50% of children are infected with RSV in the first year of life, and even 100% of children under 3 years of age [42,43]. This effect is due to an incomplete protective immune response of the host against RSV, as the virus can impair the development of effector and memory CD8+ T cells in the lung [44,45,46].

IFN-γ has important antiviral activity and is associated with the differentiation of naïve T cells into Th1 or Th2 cells [47]. Reduced interferon-γ cytokine levels have been detected in airway samples from infants with severe RSV disease [48]. Similarly, a negative correlation was found between IFN-γ mRNA levels and severity of RSV disease in nasopharyngeal samples [49], indicating a suppressed type II IFN (IFN-γ) response. In blood, the data are conflicting, with several studies finding either a positive association, a negative association or no association [50].

We performed for the first time an IFN score analysis for the type II IFN signature in NPS and peripheral blood. We found a statistically significant decrease of the type II IFN score in the peripheral blood of infants with RSV bronchiolitis. We did not attempt to find a statistical difference in the NPS, although the same trend shown in the blood is maintained. We have not shown a correlation between the type II IFN score measured in the NPS and in the peripheral blood, whereas Lopez and colleagues have recently demonstrated a correlation between IFN type I/III in patients with SARS-CoV-2 infection [51]. This is probably related to the fact that IFN-I and IFN-III are involved in the first line of defense against IFN type-II infections [52]. All of the infants studied were hospitalized with a first LRTI, were of similar age, had no family history of atopy and were therefore comparable to healthy controls. Several studies have reported a reduction in the IFN type II plasma level in patients with severe COVID-19 and RSV infection, which is comparable to our results [18,53] indicating a suppressed type II IFN (IFN-γ) response. A number of immune cell analyses suggest that IFN-γ-producing CD4+ T, CD8+ T and NK cells are depleted in patients with severe COVID-19 [54,55], which could plausibly explain the reduced IFN-γ plasma levels in ICU patients. An inadequate immune response can result from the immaturity of the neonate’s immune system. It is known that the ability to generate responses in infancy is heterogeneous in the normal population. The importance of age during the first RSV infection in determining the subsequent pattern of T-cells responses upon reinfection has recently been demonstrated in a mouse model. Neonatal RSV infection resulted in a more severe disease and strong type II cytokine responses upon reinfection, whereas delayed RSV infection resulted in increased production and less severe disease upon reinfection. These results suggest that early neonatal RSV infection may induce a long-lasting tendency for type II immune responses upon reinfection, emphasizing the importance of early infections in determining subsequent disease progression [53]. Joshi et al. showed that absolute levels of IFN-γ mRNA levels were also lower compared to infants in whom another virus or no virus was identified as the cause of respiratory symptoms. This suggests a suppression of Th1 cytokine responses at the airway level during RSV infections [56]. It would also suggest that other viral infections, particularly rhinovirus, upregulate production during acute infections. Lower levels of IFN-γ mRNA measured during RSV infection could favor the development of asthma: Renzi et al. [27] found that in children hospitalized with acute RSV infection, those who developed asthma had significantly lower levels of IFN-γ produced by their PBMCs at the time of acute RSV infection than those who did not have asthma [57].

The discrepancy between the data obtained in blood and NPS is probably due to the nature of the biological sample. In the case of IFN type II, the NPS swab (mucosal defenses) returns to normal expression before the peripheral blood. In fact, all the infants tested recovered positively from the infections a few days later. The type II IFN value measured in the NPS could be used as a biomarker indicator for the severity of infections, as a rapid return to normal levels of expression of type II ISGs indicate the end of the disease. Studies on cytokine response have produced conflicting results, probably due to the large heterogeneity in study design and sample size. Although the data suggest predominantly decreased IFN-γ production in nasal samples, the data in blood are conflicting, showing either a positive association [58], a negative association [53] or a lack of association in several studies [50]. Compared to the type II IFN score measured in peripheral blood, the score measured on an NPS result is not invasive and could reflect the real stage of infection as a marker for resolution of RSV infection in the individual infant. This biomarker is even more useful considering the fact that sampling in the case of a viral infection is often difficult to identify and standardize. How many days ago did the symptoms begin? When did the infant have access to the hospital? It is difficult to standardize the time of blood or swab sampling and testing.

However, the temporal sequence of events (low IFN-g levels predisposing to RSV infection and/or RSV infection worsening the ongoing immune response) in the individual infant can only be determined by a prospective study, which, due to the low incidence of severe LRTI in infants, would need to include a considerable number of newborns for the results to be sufficiently meaningful. The usefulness of the NPS type II IFN score as a rapid marker for resolution of RSV infection in the individual infant can only be determined by a prospective study, which would need to include a substantial number of newborns for the results to be sufficiently meaningful due to the low incidence of severe LRTI in infants. Th-1 cells in the first months of life and maturation of cellular immune functions are trigged by a strong stimulus of IFN-γ. An infant’s history of infection, rather than age, may therefore have a significant impact on the clinical development of respiratory disease, particularly with regard to the so-called sensitization phase in infancy.

Although the results shown in this paper are promising for improving the clinical management of bronchiolitis, they must be interpreted with caution. In conclusion, our study shows for the first time that the type II IFN score was significantly reduced in peripheral blood of infants with bronchiolitis by RSV compared to age-matched healthy controls; in the NPS swab, this resulted downregulation was not statistically significant and the type II IFN score in the NPS swab can be used as marker of resolution of infection or improvement of clinical conditions.

## Figures and Tables

**Table 1:** Characteristics of the study population in terms of age and white blood cells count.

Variable	Bronchiolitis (30) *	Healthy Controls (36) *	*p* Value ^§^
Age (days)	86 ± 84	92 ± 101	0.585
White blood cells count (cells × 10^9^/L)	9670 ± 650	8830 ± 992	0.386
Neutrophils (cells × 10^9^/L)	2834 ± 1129	3228 ± 1010	0.545
Lymphocytes (cells × 10^9^/L)	5400 ± 1998	4180 ± 1883	0.032 °
Eosinophils (cells × 10^9^/L)	302 ± 100	443 ± 80	0.310
Monocytes (cells × 10^9^/L)	1503 ± 1030	632 ± 301	<0.0001 °

* Data are reported as mean and SD. ^§^ Mann–Whitney U test. ° Statistically significant.

**Table 2:** ΔΔCt data and Interferon score obtained in blood of bronchiolitis RSV positive subjects.

CXCL9	CXCL10	Ido1	IFN	IFN Score
0.157065	8.18543	0.286978	0.164163	0.22557
0.624713	0.679134	0.553085	0.197167	0.588899
0.061826	0.16016	0.069976	0.311058	0.115068
0.931124	2.576414	1.962246	0.549406	1.446685
0.587774	2.936548	4.420878	0.804047	1.870297
1.766501	3.628207	1.800975	0.693805	1.783738
0.302649	1.540725	1.108899	2.195808	1.324812
0.58293	1.376756	1.961242	0.326026	0.979843
0.937127	35.02627	1.534166	0.776836	1.235647
0.728682	143.6117	12.19011	1.500456	6.845283
0.09159	25.39361	2.204072	0.203047	1.203559
0.660717	51.14568	3.818783	0.229422	2.23975
0.068105	0.242614	0.109202	0.234683	0.171942
0.127123	0.490703	0.448894	0.170006	0.30945
0.457043	9.124063	0.856635	0.262696	0.656839
1.638153	6.903458	0.736292	0.049596	1.187223
2.700755	6.703653	0.173626	1.332774	2.016765
0.282434	0.369647	0.176321	0.295971	0.289203
0.470063	14.85163	1.255824	1.693257	1.474541
0.802751	270.6202	3.105014	0.606671	1.953883
0.229872	3.890983	0.424244	0.382459	0.403351
0.693212	4.8035	0.830444	0.321862	0.761828
0.159718	0.358076	0.063926	0.44009	0.258897
0.372456	1.831377	0.710723	0.885488	0.798105
1.423442	1.934601	0.320828	1.820754	1.622098
0.276544	1.094884	0.69411	0.886863	0.790487
0.189038	0.331033	0.059418	0.266275	0.227656
0.060295	0.15324	0.119145	0.968841	0.136193
0.174432	0.771439	0.319235	0.974291	0.545337
0.45667	13.39745	0.651593	0.98962	0.820607

**Table 3:** ΔΔCt data and Interferon score obtained in blood of healthy subjects.

CXCL9	CXCL10	Ido1	IFN	IFN Score
0.927221	5.927591	2.814915	1.059956	1.937436
0.807148	0.578726	1.007283	0.800781	0.803965
0.961704	2.670438	0.346059	0.938156	0.94993
0.553646	3.473331	2.624912	1.69073	2.157821
0.558547	3.529864	1.485558	0.880558	1.183058
1.166531	6.642831	3.234286	1.094232	2.200409
0.574403	0.930456	0.491075	0.977094	0.752429
0.703173	3.167205	2.206281	1.306828	1.756554
0.593752	2.681653	2.472985	1.178443	1.825714
0.526364	2.241326	2.699156	0.94144	1.591383
0.789334	16.15611	1.675641	0.349211	1.232488
0.449238	1.028112	1.120321	2.891309	1.074216
3.026584	33.28678	3.15599	4.782118	3.969054
1.415051	6.074084	2.684512	1.954509	2.31951
1.233968	5.30949	3.012727	2.855546	2.934137
1.818892	54.26082	2.671744	1.829073	2.250408
1.482601	7.572696	3.070401	5.337788	4.204095
0.198741	1.123516	7.019939	1.755256	1.439386
0.246297	2.135114	2.707466	1.617702	1.876408
0.315997	3.798261	1.361492	0.123542	0.838745
0.547174	2.802106	3.099739	2.73358	2.767843
0.299992	31.17147	14.73604	5.374277	10.05516
0.62112	1.344691	3.800596	1.08666	1.215676
0.927419	12.62865	9.230163	1.47954	5.354851
0.525293	3.374039	2.958201	2.699701	2.828951
0.473346	7.375175	1.825008	1.997246	1.911127
0.831074	5.225671	0.523769	1.607206	1.21914
0.667976	1.468509	0.821468	0.810344	0.815906
1.640676	11.53084	1.537516	2.962262	2.301469
4.472612	44.21708	10.61164	8.757177	9.684408
0.422769	1.28918	1.027396	2.246802	1.158288
0.554591	2.148308	2.335841	0.387631	1.35145
4.242832	20.40956	2.606556	4.880706	4.561769
0.927105	3.324605	0.747185	3.002083	1.964594
1.444508	2.796676	1.462326	3.054695	2.129501
0.192616	0.748958	0.230515	1.446799	0.489737

**Table 4:** ΔΔCt data and Interferon score obtained in NPS of bronchiolitis RSV positive subjects. // date not available.

IFNg	CXCL9	CXCL10	IDO1	IFN Score
1.685659	0.106575	0.060205	0.190854	0.177204
0.199817	0.450834	0.357298	0.520083	0.450208
0.6063	0.114727	0.115976	2.218294	0.847529
2.364469	1.466056	1.387023	3.230586	1.915262
1.629892	0.515913	0.334127	1.168483	0.884869
2.218103	0.494326	0.552003	2.591719	2.211611
0.742426	3.30203	7.521585	61.60443	5.878039
0.288555	0.349889	0.12762	0.980016	0.319222
0.937276	0.708254	0.318651	3.882854	0.822765
2.629424	3.740852	2.294862	25.64459	4.465884
8.459833	0.68337	2.678824	24.66566	3.105226
0.116979	0.333878	0.191974	0.371062	0.262926
1.939566	1.338197	0.955549	12.77733	1.638882
4.688201	0.820236	1.056642	30.713	3.659446
//	0.711009	0.875694	7.346527	2.825088
0.042141	0.051571	0.025111	0.01295	0.046856
0.357725	0.264255	0.155025	3.177838	0.36583
2.861291	1.714959	2.135358	14.25268	2.485898
2.379219	0.3004	0.203204	2.950032	0.754847
0.989339	2.618225	0.873043	3.202769	2.563137
0.05656	0.025731	0.016338	0.130882	0.062945
0.590727	1.039541	0.494817	1.226243	0.764687
//	1.674171	0.778514	2.732303	1.142535
1.114745	0.356293	1.091995	18.2477	1.293056
3.358902	1.725384	1.169357	2.415619	2.123589
//	//	//	7.065799	3.783915
0.033014	0.017777	0.028004	2.806203	0.071856
3.286032	1.858926	9.757487	45.052	4.096859
//	//	//	2.414923	1.316748
//	0.060658	0.041742	//	0.052629
//	0.159294	0.03838	1.285106	0.159294
2.72743	1.535855	0.419061	1.133716	0.93273
1.111062	0.075519	0.066413	//	0.252402
0.228895	0.020628	0.01378	1.211771	0.148608
0.772693	0.185458	//	0.599208	0.599208
1.345381	0.09437	0.119168	//	0.125718
3.47402	0.311783	0.234453	2.285236	0.536745
//	1.710044	0.532687	1.712725	1.711384
//	//	0.124275	4.711086	0.617166
//	//	0.164969	4.244882	0.364803
//	0.272361	1.167929	6.361056	0.878366
//	0.672684	0.285985	0.403993	0.403993
//	//	0.262111	//	0.229
//	//	0.08179	//	0.134591
1.749853	0.583862	0.332689	0.660082	0.83433

**Table 5:** ΔΔCt data and Interferon score obtained in NPS of healthy subjects. // date not available.

IFNg	CXCL9	CXCL10	IDO1	IFN Score
0.119396	0.365331	0.212938	0.454007	0.351945
//	//	//	4.354237	3.21977
2.396478	3.402945	11.74381	21.757	4.622528
1.084336	0.501719	0.324502	0.174045	0.396846
1.370674	1.186268	0.581905	0.50408	0.732686
1.941057	1.744747	0.761611	0.479918	0.777233
0.900624	1.081239	0.490691	0.45876	0.530976
//	0.067341	0.494851	//	0.494851
0.570475	0.233015	0.373023	0.449391	0.449391
//	6.473846	7.796893	//	4.625882
4.727643	4.158529	2.109328	//	3.227281
0.478495	0.471916	1.254819	3.87319	0.919472
0.12972	0.0641	0.110914	2.88078	0.199274
3.129895	1.368325	1.13171	1.313043	1.222377
0.485004	1.065	0.874331	1.552961	1.132574
0.78607	1.009494	0.807926	0.295926	0.7852
1.857269	1.117105	0.639395	0.215011	0.936784
0.320975	1.104835	3.47792	3.986919	1.145811
0.84222	1.283846	0.833621	1.326317	1.063033
1.786934	3.155041	2.117417	0.710685	1.805562
7.505436	7.622924	4.274014	0.492337	4.072474

## Data Availability

The data presented in this study are available on request from the corresponding author.
